# Brucellosis in healthcare settings: research advances in nosocomial infection prevention and control

**DOI:** 10.3389/fpubh.2026.1859809

**Published:** 2026-08-05

**Authors:** Xuewei Du, Li Li, Guilan Wang

**Affiliations:** 1 Department of Public Health, Affiliated Hospital of Inner Mongolia Medical University, Hohhot, China; 2 Department of Infectious Diseases, Affiliated Hospital of Inner Mongolia Medical University, Hohhot, China

**Keywords:** biosafety, brucellosis, healthcare workers, infection prevention and control, knowledge attitudes and practices, laboratory-acquired infection, nosocomial infection, occupational exposure

## Abstract

Brucellosis, a globally significant zoonosis, poses substantial but often underappreciated risks within healthcare and allied professional environments. This narrative review synthesizes current research advances in the prevention and control of nosocomial and occupational brucellosis, addressing a critical gap in the existing literature that has predominantly focused on community-acquired transmission. We examine seven key dimensions: laboratory-acquired brucellosis and biosafety lapses; occupational exposure risks among healthcare workers, veterinarians, and abattoir personnel; epidemiology of hospital-associated outbreaks; standard precautions and infection control practices; risk assessment and mathematical modeling; knowledge, attitudes, and practices among at-risk personnel; and policy frameworks. Laboratory-acquired infections remain prevalent due to inadequate biosafety practices and misidentification of Brucella species. Effective prevention requires multi-layered engineering controls, administrative policies, personal protective equipment, and robust exposure management.

## 1 Introduction: brucellosis as a zoonotic public health concern and nosocomial risk

Brucellosis persists as a significant global zoonosis with profound implications for public health, especially in endemic regions across Asia, Africa, the Middle East, and parts of Latin America (1, 2). A recent evidence-based modeling study estimated the annual global incidence of human brucellosis at approximately 2.1 million cases, substantially higher than the previously assumed 500,000 cases per year, with Africa and Asia sustaining the majority of the global disease burden (3, 4). In China alone, 687,529 human brucellosis cases were reported between 2004 and 2021 (5), corresponding to an average annual reported incidence rate of approximately 2.8 cases per 100,000 individuals (5), and recent surveillance data indicate that brucellosis remains among the most prevalent zoonoses in China (6), with incidence continuing to rise in inland regions (7, 8). The disease imposes economic burdens on the livestock sector and is still a challenge for healthcare systems due to its non-specific clinical presentation, potential for chronicity, and the risk of severe complications (9, 10). The primary transmission routes for humans traditionally involve direct contact with infected animals or their birthing products, or the consumption of contaminated, unpasteurized dairy products (11, 12). However, beyond these community-acquired transmission routes, brucellosis presents a distinct and often underestimated threat within healthcare and allied professional environments. Nosocomial and occupational brucellosis, originating from laboratory, hospital, abattoir, and veterinary settings, is a key aspect of the disease epidemiology that demands dedicated prevention and control strategies (13). The re-emergence and geographic expansion of brucellosis recently have exacerbated these risks (1). Factors such as increased international travel and trade, nomadic lifestyles, and the breakdown of veterinary control programs in some regions have contributed to its spread (2, 13). In Xinjiang, China, 78,689 cases were reported between 1957 and 2023, with incidence rising from 1.77 to 36.08 per 100,000 and affected counties expanding from 21 to 100 (14, 15). Consequently, healthcare workers, laboratory personnel, veterinarians, and workers in the meat-processing industry in both endemic and non-endemic countries face potential exposure. A systematic review and meta-analysis identified that farmers, laboratory workers, and abattoir workers have 3.47 times higher odds of Brucella infection compared to the general population (16). The insidious onset of the disease, combined with variable laboratory findings leading to frequent misdiagnosis, can result in delayed recognition within healthcare facilities and subsequent accidental exposures (17). A recent analysis of 581 human brucellosis cases in Xinjiang found a blood culture positivity rate of only 4.48%, underscoring the diagnostic challenges that contribute to delayed recognition (18). This suggests that infection prevention and control (IPC) requires a strong, multi-faceted approach that extends beyond agricultural and food safety measures to genuinely incorporate the healthcare sector. This review synthesizes recent research on the epidemiology, risk factors, and control strategies for brucellosis in healthcare and allied occupational settings, without addressing clinical diagnosis and treatment aspects.

The scope of this review was deliberately defined to address the most critical and least synthesized aspects of nosocomial and occupational brucellosis. Clinical diagnosis and therapeutic management were excluded, as these topics have been comprehensively covered in numerous recent systematic reviews and clinical practice guidelines, and their inclusion would dilute the focus on prevention and control. The selection of seven thematic dimensions was based on a preliminary scoping scan of the literature, identifying these as the key pillars of a comprehensive IPC framework. Accordingly, this review addresses the following focused question: What evidence-based IPC interventions and strategies are available to prevent nosocomial and occupational brucellosis in healthcare, laboratory, veterinary, and allied occupational settings? The intended audience includes clinicians, infectious disease specialists, biosafety officers, occupational health practitioners, public health policymakers, and researchers engaged in zoonotic disease prevention. The expected contribution is to synthesize existing evidence across seven thematic dimensions, to quantify the strength of evidence through a GRADE-adapted assessment, and to identify key gaps, especially the lack of intervention effectiveness studies, that need to be addressed to strengthen IPC policies and practices in endemic regions.

## 2 Methods

This narrative review followed general principles of systematic review methodology, adapted for a narrative synthesis approach. A systematic literature search was conducted in PubMed/MEDLINE, Scopus, and Web of Science. The search covered the period from database inception to April 19, 2026. The PubMed search strategy combined brucellosis-related terms (“Brucellosis”[MeSH Terms] OR “brucellosis”[Title/Abstract] OR “brucella infection*”[Title/Abstract] OR “malta fever”[Title/Abstract] OR “undulant fever”[Title/Abstract]) with healthcare environment and occupational risk terms, organized into four conceptual modules: nosocomial infection epidemiology (combined with “Cross Infection”[MeSH Terms], “hospital infection*,” “nosocomial infection*,” “healthcare-associated infection*”); infection control and prevention (combined with “Infection Control”[MeSH Terms], “prevention and control,” “preventive measure*,” “control measure*”); healthcare setting outbreaks (combined with “Disease Outbreaks”[MeSH Terms], “outbreak*,” “epidemic*,” and “hospitals”[MeSH Terms], “hospital,” “nosocomial,” “healthcare facility*”); guidelines, policy, and education (combined with “Guidelines as Topic”[MeSH Terms], “Health Policy”[MeSH Terms], “Education”[MeSH Terms] and infection control/prevention terms). Studies focusing on diagnosis or treatment were excluded using the NOT operator. Equivalent terms were used for Scopus and Web of Science searches, adapted to each database syntax. Reference lists of included articles and relevant systematic reviews were also screened to identify additional eligible studies.

**Figure 1:**
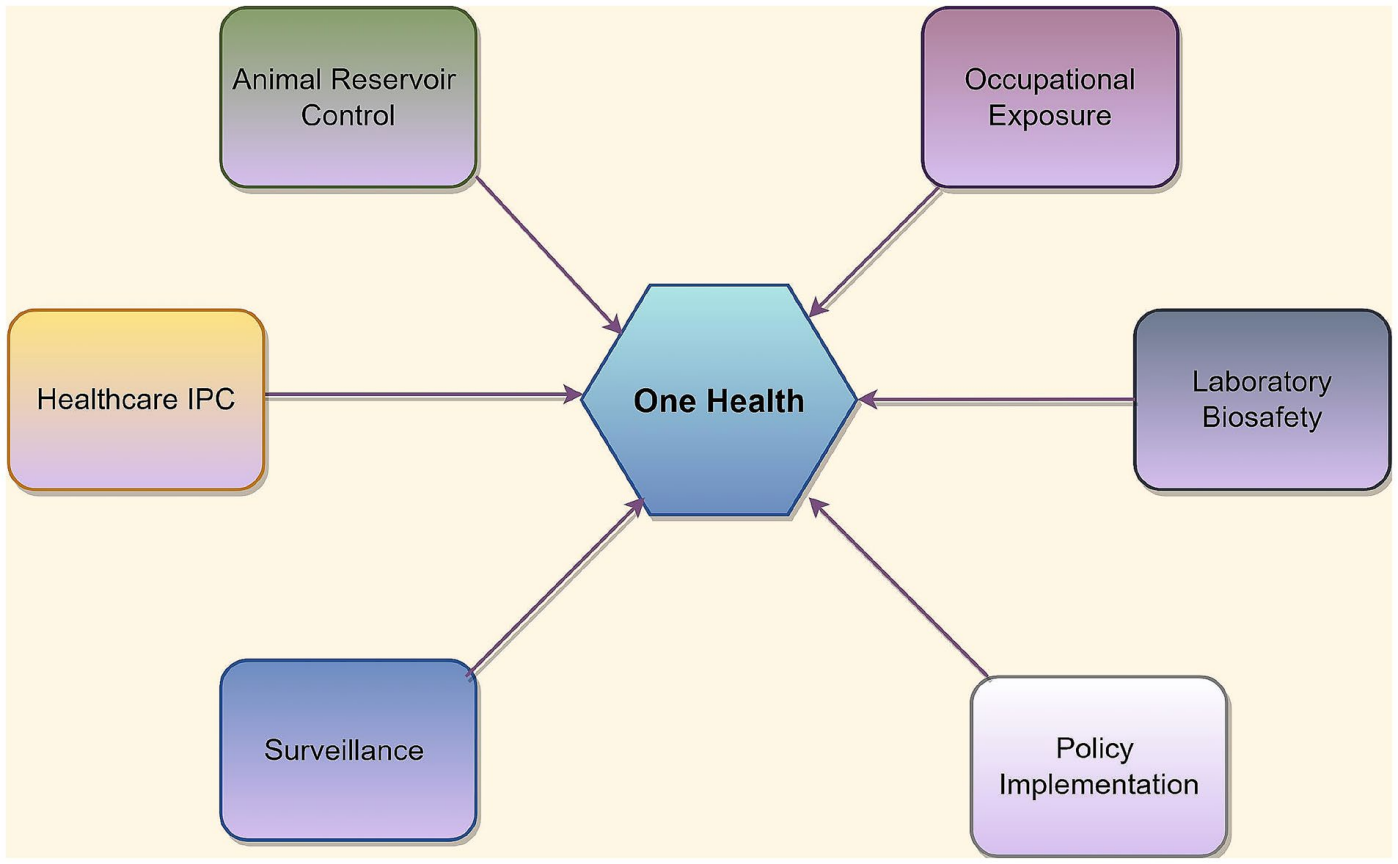
Proposed one health IPC framework for brucellosis prevention. The framework integrates six key components around a central One Health coordination hub: Animal reservoir control, occupational exposure reduction, laboratory biosafety, healthcare IPC, surveillance, and policy implementation. Bidirectional arrows indicate the flow of information and actions between each component and the coordinating center.

Inclusion criteria were as follows. Studies were included if they addressed brucellosis in healthcare, laboratory, veterinary, or other occupational settings; reported on at least one of the seven thematic dimensions; were published in English; and employed observational, experimental, modeling, or policy analysis methods. Exclusion criteria were studies specifically focused on community-acquired brucellosis, clinical diagnosis, or therapeutic management; and case reports of individual community-acquired infections without relevance to nosocomial or occupational prevention. The initial database search yielded 1,071 records from PubMed/MEDLINE, 229 from Scopus, and 1,675 from Web of Science. After removing 912 duplicate records, 2,063 records underwent title and abstract screening, of which 108 proceeded to full-text review. Following full-text assessment, 54 studies were included in the narrative synthesis (Figure 1). The screening and selection process followed PRISMA guidelines (19).

Two authors collaboratively screened all titles and abstracts, then jointly reviewed full texts against the inclusion criteria. Although screening was not conducted by two independent reviewers in the strict systematic review sense, all included studies were discussed and consensus was reached before inclusion. A structured data extraction form was used to record study characteristics, design, setting, population, and key findings. This review was not registered in PROSPERO, as it was conducted as a structured narrative review rather than a full systematic review. We acknowledge that the absence of dual independent screening is a methodological limitation.

A GRADE-adapted approach was used to assess the certainty of evidence for each thematic dimension (20, 21). Initial certainty was determined by study design: randomized controlled trials started as high certainty, and observational studies started as low certainty. Certainty was then assessed across five GRADE domains: risk of bias, inconsistency, indirectness, imprecision, and publication bias. For observational studies, certainty could be upgraded if there was a large effect size, evidence of a dose–response relationship, or if all plausible confounders would reduce the observed effect.

The application of GRADE to thematic dimensions rather than specific outcomes is supported by Murad et al. (21), who provided guidance on rating certainty in the absence of a single estimate of effect. This approach is appropriate for narrative syntheses across heterogeneous study designs. To enhance transparency, Table 1 details how each GRADE domain was assessed for each evidence claim.

**Table 1:** GRADE evidence profile for brucellosis IPC thematic dimensions.

Thematic dimension	Risk of bias	Inconsistency	Indirectness	Imprecision	Publication bias	Factors affecting certainty	Final certainty
1. Laboratory-acquired brucellosis	Serious (case reports/series)	Not serious	Not serious	Serious	Likely	Upgraded: large effect (outbreaks despite BSL-2); dose–response	Moderate
2. Occupational exposure risks	Serious (observational)	Not serious	Serious	Not serious	Undetected	Upgraded: large effect (pooled OR 3.47); consistency across populations	Moderate
3. Hospital-associated outbreaks	Serious (descriptive only)	Not serious	Serious	Serious	Likely	Not upgraded: no analytical designs; high risk of bias	Low
4. Standard precautions and IPC	Serious (expert guidelines)	Not serious	Serious	Serious	Possible	Not upgraded: no controlled evaluations; compliance surveys only	Low
5. Risk assessment and modeling	Serious (models not validated)	Not serious	Serious	Serious	Possible	Not upgraded: indirect evidence from veterinary contexts	Low
6. KAP among at-risk personnel	Moderate (quasi-experimental)	Serious (I2 = 99.4%)	Not serious	Serious	Possible	Upgraded: large volume (79 studies); quasi-experimental effectiveness	Moderate
7. Policy and program evaluation	Serious (program evaluations)	Not serious	Serious	Serious	Possible	Not upgraded: no healthcare IPC focus; indirect from veterinary programs	Low

GRADE, Grading of Recommendations Assessment, Development and Evaluation. Initial certainty was determined by study design (RCTs = high; observational studies = low). Certainty was then assessed across five domains: risk of bias, inconsistency, indirectness, imprecision, and publication bias. Certainty could be upgraded based on large effect size, dose–response relationship, or plausible confounding. Final certainty ranges from high to very low. IPC, infection prevention and control; KAP, knowledge, attitudes, and practices; OR, odds ratio; BSL, biosafety level.

## 3 Laboratory-acquired brucellosis: risks, outbreaks, and biosafety lessons

Laboratory-acquired brucellosis is a typical example of occupational risk in biomedical environments and a key area for IPC intervention. Brucella species, especially *B. melitensis*, are classified as Risk Group 3 pathogens due to their ability to cause severe disease via aerosol transmission and their low infectious dose. Multiple incidents of accidental exposure in clinical, research, and teaching laboratories have been documented worldwide, often with serious consequences.

Outbreak investigations have repeatedly revealed common and preventable failures in biosafety practices. A seminal report detailed an outbreak in which variable Gram stain results led to the initial misidentification of *B. melitensis*, with laboratory personnel handling cultures without adequate protection while unaware of the true risk (17). Similarly, an outbreak at a pharmaceutical factory in southeastern China was linked to manual handling of sheep placentas, inadequate disinfection, reuse of protective clothing, and aerosol dissemination (22). An analysis of public health events related to brucellosis in China from 2006 to 2019 found that failure to handle suspected Brucella samples according to recommended biosafety practices was the primary risk factor for laboratory infections in hospitals (23). A serious teaching laboratory incident at a university resulted in 28 confirmed cases, illustrating the consequences of inadequate supervision and biosafety oversight in educational settings involving veterinary students (23).

Investigations of specific incidents provide granular insights into failure points. An accidental exposure in a diagnostic laboratory was traced to a single operator manipulating Brucella isolates on an open bench, with detailed tracing revealing multiple laboratory non-compliances (24). In Thailand, a laboratory technician acquired *B. melitensis* infection, with molecular typing confirming the isolate matched the patient strain handled in the laboratory; the presumed mechanism was aerosol inhalation during photography of culture plates without adequate containment (25). These cases demonstrate that risks are not confined to deliberate culture work but also arise during procedures such as aliquoting serum, performing agglutination tests, or handling unidentified blood cultures.

Post-exposure management remains inconsistent. Surveys of clinical laboratories have revealed variability in adherence to recommended post-exposure prophylaxis protocols, with some institutions relying solely on serological monitoring while others implement antibiotic prophylaxis for high-risk exposures (24). The overall infection control strategy for laboratory brucellosis requires that all unidentified febrile blood culture isolates be treated as potential Brucella until proven otherwise; that biological safety cabinets be mandatory for aerosol-generating procedures; and that clear communication between microbiology and clinical services be maintained when brucellosis is suspected.

## 4 Occupational exposure risks

Occupational exposure to Brucella extends beyond the laboratory to encompass veterinary practice, abattoir work, and animal husbandry. A systematic review by Pereira et al. consolidated evidence from 63 studies, identifying rural workers, abattoir workers, veterinarians, laboratory workers, and hunters as the groups most exposed to occupational Brucella infection (16). A meta-analysis of 3 case–control studies yielded a pooled odds ratio of 3.47 (95% CI: 1.47–8.18) for infection among occupationally exposed groups compared to non-exposed groups (16).

Cross-sectional studies from Argentina, Tanzania, and Egypt have quantified risk factors. Molineri et al. surveyed 741 Argentinean veterinarians (75.8% response rate, n = 562) and reported a cumulative incidence of brucellosis of 29.1% (26). In Tanzania, Ntirandekura et al. found that assisting in parturition without wearing protective gear was associated with higher infection risk (OR = 5.6, *p* = 0.02) (27). Studies from China documented variable PPE use rates among occupational groups (28, 29). Recent evidence from South Africa found that less than half of 137 veterinary fieldworkers received regular occupational health training, and PPE provision was inconsistent (30). In the United Arab Emirates, a One Health study of 86 abattoir workers identified a seroprevalence of 8.1%, with butchers at greater risk (31). In China, a cross-sectional survey of 238 workers in Jiangsu Province found that 21% of occupationally exposed individuals were infected, with slaughtering associated with a relative risk of 1.80 and handwashing with a relative risk of 0.25 (28).

Abattoir workers face particular risks due to the nature of animal slaughter and processing. Outbreaks have been documented in pork packing plants in the United States, where 19% of kill-floor workers were infected (32), in Spanish meat-processing facilities (33), and in Jordanian military hospital settings (34). The investigation of these outbreaks reveals common themes: inadequate PPE, poor facility design, and insufficient training on zoonotic disease risks. Pharmacists and workers in facilities processing medicinal animal products, such as placental extracts, are also at risk, as evidenced by the pharmaceutical factory outbreak (22).

The systematic review by Pereira et al. consolidates this evidence, identifying rural workers, abattoir workers, veterinarians, laboratory workers, and hunters as the groups most exposed to occupational Brucella infection (16). The predominant risk factors across these groups include direct contact with animal fluids and tissues, failure to use PPE, accidental exposure to live vaccines (e.g., Rev-1), and non-compliance with biosafety standards (16). The frequent isolation of *B. melitensis* and *B. abortus* in occupational cases aligns with the species economic and zoonotic importance (16). This body of evidence mandates that brucellosis IPC policies must be designed with these diverse occupational landscapes in mind, requiring tailored interventions for different at-risk professional groups within and adjacent to the healthcare continuum.

## 5 Epidemiology and investigation of hospital-associated brucellosis outbreaks

Understanding the epidemiology of hospital-associated brucellosis outbreaks is central to developing effective prevention strategies. These outbreaks, while less frequent than community-acquired waves, present unique transmission dynamics and investigation challenges. They often originate from a single index case whose infection is not initially recognized, leading to secondary cases among healthcare workers or, rarely, other patients.

The source of hospital outbreaks is typically an unrecognized Brucella infection in a patient whose clinical specimens are processed in clinical laboratories. As previously noted, misidentification is a common precipitating factor (17). The subsequent exposure of laboratory personnel through aerosol-generating procedures (e.g., vortexing, centrifuging) or direct skin contact with cultures or contaminated surfaces can lead to multiple secondary cases. The outbreak at Methodist Dallas Medical Center, involving eight patients, shows the importance of standardized microbiology processes and coordinated collaboration among hospital departments and local health departments (35).

The investigation of hospital outbreaks requires a coordinated approach involving infection control teams, microbiology laboratories, occupational health services, and public health authorities (35). The outbreak was traced to index cases with no apparent epidemiological link initially, highlighting the importance of molecular typing in establishing connections between cases (24, 25). Molecular typing techniques, such as 16S-23S rRNA intergenic spacer region analysis and multiple-locus variable-number tandem repeat analysis (MLVA), have become key tools for confirming laboratory-acquired infections and identifying the source of exposure (25).

Geographic patterns of hospital-associated brucellosis reflect the underlying epidemiology of the disease. In endemic regions, hospital outbreaks may be underreported due to the high background incidence of community-acquired cases, making it difficult to distinguish nosocomial transmission. In non-endemic regions, hospital outbreaks are more likely to be recognized and investigated, but they often involve imported cases or laboratory exposures, necessitating region-specific preventive emphases (23). Seasonality, often observed in community brucellosis with peaks during the animal breeding season, may indirectly increase the risk of laboratory exposure by increasing the volume of suspicious specimens being processed (36).

## 6 Standard precautions and infection control practices

Standard precautions for brucellosis IPC encompass engineering controls, administrative policies, and personal protective equipment. Engineering controls include biological safety cabinets (BSCs), sealed centrifuge rotors, and facility design that separates high-risk areas. Administrative controls include standard operating procedures, training mandates, and specimen notification protocols. PPE requirements vary by task but generally include gloves, gowns, masks, and eye protection for procedures with potential for splash or aerosol generation.

Compliance with these measures remains variable. Wang et al. reported that among occupational groups in China, PPE use rates remained low (20.13%), with significant disparities based on occupation, education, and region; glove use was 38.26% and mask use was 31.80% (29). Gloves, masks, rubber boots, and work clothes were the most commonly used items, but compliance with more specialized equipment such as respirators was lower. This variability in compliance shows the need for targeted interventions to improve PPE use among high-risk occupational groups.

Environmental decontamination is another important component of standard precautions. Brucella species can survive in the environment for extended periods, especially in cool, moist conditions. Effective decontamination requires appropriate disinfectants (e.g., 70% ethanol, sodium hypochlorite, glutaraldehyde) and protocols for cleaning spills of potentially infectious materials. The pharmaceutical factory outbreak highlighted the consequences of insufficient disinfection practices (22).

A comprehensive infection control strategy for brucellosis requires integrating these standard precautions with risk assessment, staff training, and post-exposure management. Clearly labeling specimens from suspected brucellosis cases, communicating risks to laboratory personnel, and establishing clear protocols for handling unidentified blood cultures are key elements of a comprehensive IPC program (24). The investigation of specific incidents provides granular insights into failure points, such as manipulating Brucella isolates on an open bench or photographing culture plates without containment (24, 25). These cases underscore that risks are not confined to deliberate culture work but also arise during procedures like aliquoting serum, performing agglutination tests, or handling unidentified blood cultures, emphasizing the need for standard precautions to be applied consistently across all laboratory activities.

## 7 Risk assessment and mathematical modeling for IPC planning

Risk assessment and mathematical modeling have emerged as valuable tools for brucellosis IPC planning, allowing for the quantification of transmission risks and the evaluation of control strategies. Dalrymple developed a model for assessing the risk of introducing brucellosis into a brucellosis-free area, providing a framework for gathering and analyzing data to estimate the risk associated with proposed changes in control programs (37).

Roy et al. introduced a network control theory approach for modeling brucellosis transmission in sub-Saharan Africa, demonstrating how resources can be optimally distributed among vaccination, surveillance, culling, and pasteurization (38). Havas et al. developed an agent-based model for the Kakheti region of Georgia, finding that reduction in disease in humans was best accomplished through control in the most commonly owned animal species (39).

A systematic review of dynamic modeling studies in China identified 49 articles, with 22 utilizing empirical data and 27 relying on theoretical constructs (40). The review noted that limitations of existing models include lack of heterogeneity in research and absence of results at the herd/flock scale, primarily due to lack of relevant data. These modeling efforts indicate that effective IPC for brucellosis often requires an integrated One Health approach, with control of the animal reservoir being the most sustainable way to reduce human risk (38, 39, 41).

Risk modeling has also been applied to specific occupational and environmental interfaces. A citizen-science informed risk model for bovine brucellosis transmission from elk to cattle in the Greater Yellowstone Area helped estimate the expected frequency of cattle outbreaks and evaluate the potential effectiveness of various management strategies like hazing elk or altering cattle grazing schedules (42). From an economic perspective, models assessing cattle producers incentives for brucellosis prevention under uncertainty calculate the breakeven effectiveness levels required for various on-farm biosecurity measures to be cost-effective, guiding policy on potential cost-sharing programs (43).

These modeling efforts underscore a critical insight: effective IPC for brucellosis, including in occupational settings, often requires an integrated, One Health approach. Models that incorporate human, animal, and environmental components demonstrate that controlling the animal reservoir is the most sustainable way to reduce human risk (38, 39). For hospital and laboratory IPC specifically, risk modeling can help prioritize investments in biosafety infrastructure, training programs, and vaccine stockpiles for post-exposure prophylaxis based on the local prevalence of disease and the volume of high-risk work being conducted.

## 8 Health education, knowledge, attitudes, and practices among at-risk personnel

The human factor is paramount in infection control. Even the strongest engineering controls and policies can fail if the knowledge, attitudes, and practices (KAP) of at-risk personnel are inadequate. Research consistently reveals significant gaps in brucellosis-related KAP among high-risk groups, which directly correlates with risky behaviors and increased infection rates.

Systematic reviews indicate a global deficit in brucellosis awareness and knowledge. A meta-analysis of 79 observational studies found a pooled awareness level of only 55.5% among studied populations, with knowledge about transmission modes and clinical signs being even lower (44). While health workers and veterinarians generally possess greater knowledge, livestock owners, farmers, abattoir workers, and the general public in endemic regions exhibit alarmingly low levels of understanding (44, 45). A study in Tajikistan found 85% of small-scale dairy farmers had never heard of brucellosis (45), and similar findings are reported from Nigeria, Sri Lanka, and other endemic regions (46, 47).

This lack of knowledge directly enables high-risk practices. Consumption of unpasteurized dairy products and direct contact with aborted materials are widespread (11, 45, 48, 49). In occupational settings, studies from China documented variable PPE use rates among abattoir workers and abattoir employees (29). Knowledge appears to be a necessary but insufficient driver of behavioral change; self-efficacy and cues to action are also important determinants of preventive behavior, with effect sizes comparable to that of knowledge itself, and high-risk practices persist even among those aware of the disease (45). Conversely, a study in Egypt demonstrated that applying protective measures significantly reduced infection risk (11).

Educational interventions grounded in behavioral theory have shown promise in promoting protective behaviors among at-risk communities and students (50–53). For example, the PRECEDE-PROCEED model has been applied to livestock vaccination behaviors (52), and the Health Belief Model has been used to design interventions for marginalized women in Iran, where cues to action effectively promoted brucellosis-preventive behaviors (50). These interventions typically combine knowledge dissemination with strategies to enhance self-efficacy and address perceived barriers to preventive behaviors.

However, the sustainability of these educational interventions remains a concern. Most studies report only short-term improvements in knowledge and behaviors, with limited long-term follow-up. Also, the translation of improved KAP into reduced infection rates has not been demonstrated. Health education programs must be integrated with broader IPC strategies, including regulatory frameworks and infrastructure improvements, and should leverage community networks and agricultural extension services to create consistent messaging (54, 55). As evidenced by the Israeli experience, the socio-political contexts that shape health behaviors must also be considered (56, 57).

## 9 Policy, regulation, and program evaluation frameworks

International standards for brucellosis prevention and management provide a framework for national programs (58). However, the translation of these standards into effective national policies remains challenging, especially in endemic, low-income countries (10, 59). The collapse of veterinary infrastructure in some former Soviet republics has contributed to brucellosis re-emergence (13).

National control programs typically combine veterinary measures (e.g., vaccination, test-and-slaughter) with public health components like food safety regulations and health education. The success of these programs significantly impacts occupational risk. Greece differentiation between vaccination zones on the mainland and eradication zones on the islands showed a significantly higher human incidence in the vaccination zones, prompting calls for re-evaluation of animal control strategies (60). Successful eradication in specific regions, such as the 10th Region of Chile or the Republic of Korea, shows that intensive, well-managed programs based on test-and-slaughter, movement controls, and vaccination can drastically reduce prevalence in livestock and, so, human cases (61, 62). Economic analyses from Brazil, India, and other countries generally show that vaccination programs, and sometimes combined strategies, are cost-effective investments for society (63–65).

From a strict healthcare IPC perspective, policies must mandate several key elements. First, brucellosis should be a notifiable disease for both public health and occupational health authorities, ensuring timely detection of outbreaks and exposures (1). Second, national guidelines for clinical laboratory biosafety and for the management of suspected Brucella samples must be established and enforced (23). Third, occupational health regulations should recognize brucellosis as a significant risk for specific professions (veterinarians, abattoir workers, laboratory staff) and require employers to provide appropriate PPE, training, and access to medical surveillance or post-exposure management (16, 66).

Program evaluation is key to measure impact and identify weaknesses. The evaluation of national programs reveals both successes and persistent challenges. In Kazakhstan, which reports over 1,300 human cases annually (7.6 per 100,000 inhabitants), a One Health evaluation identified critical gaps in interdisciplinary collaboration, data sharing between veterinary and public health sectors, and stakeholder engagement that hindered control efforts (67). In Greece, analysis of 1,786 notifications over a 16-year period (2005–2020) revealed a statistically significant decreasing trend (*p* < 0.001), but also showed that vaccination zones on the mainland had significantly higher human incidence than eradication zones on the islands, prompting calls for re-evaluation of animal control strategies (60). The Republic of Korea achieved successful eradication through intensive test-and-slaughter programs combined with movement controls (61), while Chile 10th Region demonstrated that well-managed programs can drastically reduce both livestock prevalence and human cases (62). These contrasting outcomes highlight that program design, sustained funding, and adaptive management based on continuous evaluation are critical determinants of success. Similarly, analyzing the reasons for unsuccessful programs points to common themes: inadequate funding, lack of intersectoral coordination, and failure to sustain interventions over time. A scoping review of brucellosis in Armenia identified only 19 relevant studies over four decades, highlighting persistent knowledge gaps in endemic regions (68). In France, an OASIS evaluation of the laboratory diagnostic surveillance system found that while the right people and techniques were in place, system use remained imperfect (69).

## 10 Discussion

### 10.1 Critical appraisal of included evidence

The evidence base for brucellosis IPC is constrained by methodological heterogeneity. Pereira et al. (16) synthesized 63 studies spanning 1962–2018, yet only 3 case–control studies met criteria for pooled analysis. Zhang et al. (44) encountered I^2^ = 99.4% heterogeneity across 79 KAP studies, limiting the precision of the pooled 55.5% awareness estimate. This heterogeneity reflects genuine variability in study populations, designs, and endemicity contexts rather than methodological deficiency alone.

Selection bias affects the cross-sectional literature. Molineri et al. (26) achieved a 75.8% response rate (562/741), but non-responders may have differed systematically in exposure history. Ntirandekura et al. (27) sampled 156 patients presenting with malaria-like symptoms rather than the general population, potentially inflating seroprevalence estimates. These limitations warrant cautious interpretation but do not invalidate the observed associations (16, 27).

Geographic concentration of evidence is pronounced. A substantial proportion of included studies originate from China, the Middle East, and Central Asia (1, 5, 71), reflecting endemic disease burden but limiting generalizability to non-endemic settings. European and North American literature consists predominantly of outbreak investigations rather than systematic surveillance, creating a publication bias toward incident cases over baseline risk.

Modeling studies (37–40) provide theoretical frameworks for control, but their empirical validation remains limited. Of 49 dynamic modeling articles identified in China, only 22 utilized empirical data, and none incorporated healthcare-specific transmission parameters (40). This gap between theoretical models and empirical evidence weakens the foundation for evidence-based IPC planning.

The observational nature of the majority of included evidence means that identified risk factors represent associations rather than established causal relationships. For example, the OR = 5.6 for assisting parturition (27) indicates association, but residual confounding by overall hygiene practices or socioeconomic factors cannot be excluded. Policy recommendations derived from observational evidence should therefore be considered expert-informed proposals rather than evidence-based conclusions.

### 10.2 Policy recommendations: expert-informed proposals

Given these evidence gaps, the policy framework presented here should be interpreted as expert-informed proposals rather than evidence-based recommendations (1, 9, 11). The phased implementation framework, mandatory reporting provisions, and funding mechanisms draw on the collective experience of national programs (6, 58) but have not been directly tested in healthcare settings. No study evaluated the effectiveness of integrated IPC programs in reducing nosocomial brucellosis incidence.

### 10.3 Healthcare-specific evidence gaps and future research directions

The scarcity of healthcare-specific IPC intervention trials reflects structural constraints (16, 44, 68). First, ethical considerations preclude deliberate exposure of workers to Brucella. Second, the unpredictable occurrence of outbreaks complicates prospective randomized designs. Third, the low annual incidence at any single institution necessitates multi-center collaboration. Fourth, sustained interdisciplinary coordination across veterinary, public health, and occupational health sectors is difficult to maintain over the follow-up periods required to detect intervention effects.

Feasible study designs for future research include: (1) stepped-wedge cluster randomized trials evaluating phased biosafety protocol implementation; (2) prospective cohort studies with extended follow-up in high-volume diagnostic laboratories; (3) quasi-experimental before-after studies of PPE training programs; (4) simulation modeling to estimate intervention impact (38, 39); and (5) mixed-methods evaluations integrating IPC indicators into national brucellosis control programs (67). A coordinated research agenda across these designs would address the most critical evidence gaps (1, 6, 68).

### 10.4 Limitations

Several limitations should be acknowledged. First, as a narrative review, it does not provide quantitative effect estimates, and the GRADE-adapted assessment was applied at the dimension level. Second, the search was restricted to English-language publications, potentially introducing language bias. Third, publication bias toward positive findings may overestimate intervention effectiveness. Fourth, the rapidly evolving epidemiology of brucellosis means that burden data may have changed since the search was conducted (5, 70). Fifth, the absence of dual independent screening may have introduced selection bias, although the structured thematic framework was designed to reduce this risk (12). Sixth, the policy recommendations, while grounded in the reviewed evidence, are necessarily inferential: no study directly tested the phased implementation framework proposed here (1, 6, 58).

## 11 Conclusion and future directions in nosocomial brucellosis prevention

Brucellosis remains a persistent threat to healthcare workers and allied professionals, with laboratory-acquired infections and occupational exposures in abattoirs, veterinary settings, and animal-handling industries representing a significant yet preventable burden. Recurring themes from outbreak investigations include lapses in biosafety practices, pathogen misidentification, and inadequate PPE use, which point to specific, actionable intervention targets. Prevention requires multi-layered strategies integrating engineering controls, administrative policies, PPE, and robust exposure management plans.

Critical evidence gaps persist, particularly the scarcity of healthcare-specific IPC intervention trials and the limited generalizability of evidence concentrated in endemic regions (16, 44, 68). Future research should prioritize prospective intervention studies in high-risk settings, healthcare-adapted risk models, and integration of IPC indicators into national evaluations. The policy recommendations proposed here should be evaluated through rigorous studies before widespread implementation (1, 67).
